# Contrast enhanced ultrasound evaluation of nephron sparing treatment for renal cell carcinoma

**DOI:** 10.1007/s00261-025-05314-y

**Published:** 2025-12-09

**Authors:** Richard G. Barr, Davide Roccarina, Louis Mazzareilli, Parker Bauman, Kevin Cheng

**Affiliations:** 1 Northeast Ohio Medical University, Rootstown, USA; 2 Azienda Ospedaliero-Universitaria Careggi, Florence, Italy; 3 Yale New Haven Health System, New Haven, USA

**Keywords:** Renal sparing procedures, CEUS, Radiofrequncy ablation, Microwave ablation, Partial nephrectomy, Tumor recurrence

## Abstract

**Purpose:**

To determine the accuracy of CEUS in identifying residual or recurrent tumor post nephron-sparing procedures.

**Materials and methods:**

CEUS studies performed post nephron sparing procedure (RFA, cryoablation, MWA, partial nephrectomy) from January 2018 to May 2025 were retrospectively reviewed at 2 centers. Informed consent was waived. Patient charts were reviewed for follow-up procedures and patient status. CEUS studies were performed on a Philips Epic ultrasound with a C5-1 transducer using Lumason with a dose of 1.0 to 2.0 cc. A 3-minute cine clip was saved. An examination was considered a true positive if there was contrast in the procedure site and either biopsy proven tumor or persistent enhancement for over 1 year. An examination was considered a true negative if there was no contrast enhancement in the procedure site.

**Results:**

There were 193 lesions in 188 patients. (mean age 68.0 years +/− 1.4 {SD} (range 33–90) ; 115 men). There were 16 radiofrequency ablation, 38 cryoablations, 4 microwave ablations, and 135 partial nephrectomies. The average lesion size was 2.9 cm (IQR 2.2,3.7; range 1.4–7.1 cm). There were 17 (8.8%) true positive cases and 176 (91.2%) true negative cases. Time of follow-up ranged from 1 to 336 months (mean 35.1, IQR 12, 70). There were no false positive or false negative cases based on biopsy and long-term follow-up. There were 92 (47.7%) that also had CECT. There was 96.7% concordance of CEUS with CT. There were 3 CEUS cases positive with a negative CECT.

**Conclusions:**

CEUS is an accurate method in determining residual or recurrent tumor in patients having nephron sparing procedures.

## Introduction

Renal cell carcinoma (RCC) is the most common type of renal cancer in adults, accounting for 80–85% of all primary renal neoplasms and is the 13th most common cause of cancer death worldwide [1, 2]. Nephron-sparing procedures, particularly partial nephrectomy, have become the standard of care for the management of small renal carcinomas masses, aiming to preserve renal function while achieving effective oncological control. Accurate pre-procedure imaging is critical to delineate tumor margins, assess perfusion, and guide surgical resection or image guided ablation with minimal damage to healthy renal parenchyma [3–5]. Contrast enhanced computed tomography (CECT) and contrast enhanced magnetic resonance imaging (CEMRI) have been used to assess post treatment effectiveness [6]. However, many patients have a contraindication to CT or MRI contrast due to chronic kidney disease, contrast allergy, or prior nephrectomy [7, 8]. Traditional imaging modalities such as conventional B-mode ultrasound (US) and Doppler US have limitations in sensitivity due to spatial resolution, especially in differentiating tumors from surrounding vascularized tissue.

Contrast-enhanced ultrasound (CEUS) has emerged as a valuable adjunct in nephron-sparing surgery, offering real-time, non-nephrotoxic, excellent safety profile, radiation-free visualization of renal microvascular perfusion [9, 10]. Ultrasound contrast agents are gas filled bubbles with a lipid shell, about the size of red blood cells. When excited by low mechanical index ultrasound, the bubbles resonant generating a nonlinear signal. Because the adjacent tissues provide a linear signal and can be subtracted out, the CEUS image is a contrast only image [10]. By utilizing microbubble intravascular contrast agents, CEUS enhances the detection of tumor vascularity and improves the delineation of tumor boundaries, particularly in isoechoic or complex lesions. Furthermore, CEUS can aid in intraprocedural assessment of residual tumor tissue and vascular integrity post-resection, thereby supporting precision surgery and image guided ablation optimizing functional outcomes. Ultrasound contrast is an intravascular contrast and does not extravasate. Therefore, the appearance of bubbles confirms the presents of vascularity [9–11].

To date there have been only small studies evaluating the use of CEUS for post procedure follow-up of renal masses with no substantial long-term follow-up.

The American Urologic Association guidelines recommend continued imaging follow-up after nephron sparing procedures as recurrences have occurred rarely up to 20 years post procedure [12]. As the role of minimally invasive and robotic-assisted techniques expands, the integration of CEUS into nephron-sparing procedures represents a promising advancement in enhancing surgical and interventional precision, evaluating post procedure recurrence, and preserving renal function.

This paper reports a retrospective two site study with a large number of CEUS examinations post nephron sparing procedures with long term follow-up.

## Materials and methods

### Study participants

This retrospective study was conducted at two centers. Both centers had local investigational review board approval, and the study was HIPAA compliant. Informed consent was waived by both investigational review boards.

The PACS systems were reviewed from January 2018 to May 2025 for patients having a CEUS examination during or following a nephron sparing procedure (radiofrequency ablation, cryoablation, microwave ablation, or partial nephrectomy) for renal cell carcinoma (RCC). Patients with benign lesions were excluded. Patient demographics were recorded from the medical record. The time of the CEUS examination from the nephron sparing procedure, time of follow-up studies including CEUS, CECT or CEMRI image were recorded. If additional treatment procedures were performed, these were also recorded. An examination was considered negative if no enhancement of the ablated site or in the nephrectomy bed was present. If there was contrast enhancement in the ablation site, in the nephrectomy bed or if the enhancement persisted over 1 year if no biopsy or follow-up procedure was performed, the examination was considered positive.

All renal masses were confirmed to be highly suspicious for renal malignancy by CECT, CEMRI or CEUS before the procedure. All lesions were biopsied before the procedure with the pathology recorded with one lesion biopsied by FNA confirming RCC but unable to grade the tumor. Pathology of the biopsy was performed by pathologists with greater than 5 years’ experience in evaluating renal biopsies.

### Contrast enhanced ultrasound examinations

Both centers used a Philips Epiq system (Philips Ultrasound, Bothell, WA) using a C5-1 transducer. The mechanical index (MI) was set at 0.4 or less. After a standard retroperitoneal US examination, a CEUS was performed using 0.5 to 2.0 cc of Lumason (Bracco Diagnostics, Princeton, NJ) followed by a 10 cc bolus of normal saline. The agent was activated as instructed in the package insert and administered as previously reported [13]. The CEUS examination was performed by injecting the ultrasound contrast agent through a 20 g IV placed in the antecubital fossa using a three-way stopcock [14]. A dual display was used with one image a B-mode image for localization of the procedure site and the other the contrast enhanced image. The contrast image gain before injection of contrast was set such that there was only minimal visualization of the subcutaneous tissue. Imaging started after the injection and lasted for 3 min and was recorded as a cine clip. If the examination was suboptimal a second injection was given after contrast from the first inject was no longer present (high MI was used to burst the bubbles) using the same dosage. In both sites the examinations were performed by sonographers with a minimum of 3-year experience in performing CEUS. Site 1 images were interpreted by a radiologist (RGB) with 25-years’ experience with CEUS and site 2 images were interpreted by a radiologist (LM) with 7-years’ experience in CEUS.

### Statistical analysis

Descriptive statistics were produced for the demographic characteristics of this study sample. Quantitative variables were expressed as the mean value and standard deviation (SD), and qualitative variables were summarized as counts and percentages.

## Results

**Fig. 1:**
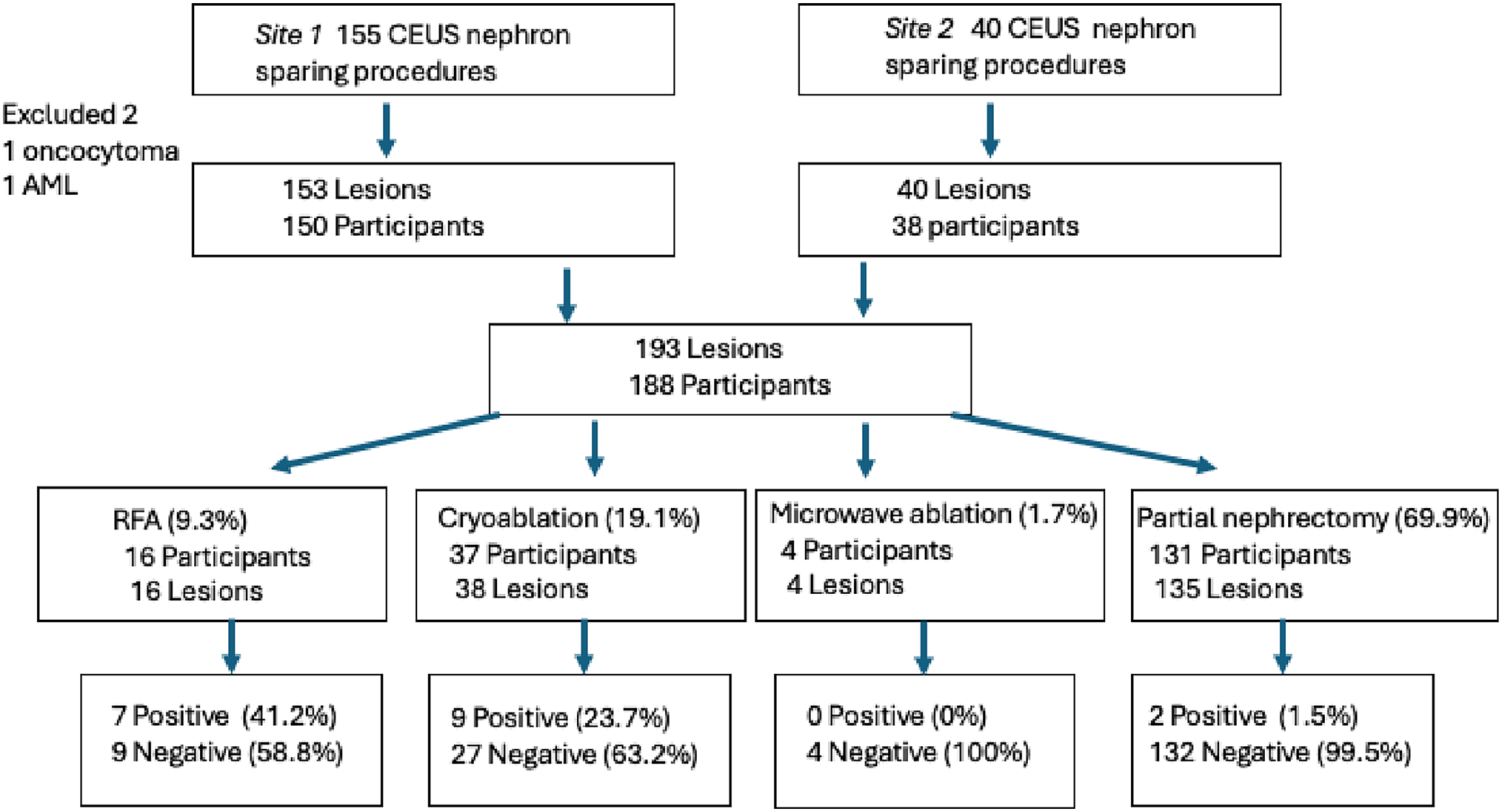
Flow chart of the study enrollment

There was a total of 193 participants, 153 from site 1 and 40 from site 2. Two cases were excluded due to benign neoplasms. All CEUS studies were adequate for diagnosis. Figure 1 is a flow chart of enrollment. Age ranged from 33 to 90 years (average 68.0 years; IQR 58.0–73.0). Participants were 78 (40.4%) females and 115 (59.6%) males. 95 (49%) tumors were in the right kidney and 98 (51%) in the left kidney (*p* = 0.807).

Tumors largest dimensions were 2.9 cm (IQR 2.2–3.7 cm: range 1.4–7.1 cm). Tumors were complex cystic 17(8.8%) or solid 176 (91.2%). Procedures included cryoablation 38 (19.7%), microwave ablation (MWA) 4 (2.1%), partial nephrectomy 135 (69.9%) and radiofrequency ablation (RFA) 16 (8.3%).

The time to follow-up with CEUS ranged from 1 to 336 months (average 35.1months; IQR 12.0, 70.0). Participant demographic, lesion size, solid or cystic, and length of follow-up for both sites are listed in Table 1. Complex cystic and solid renal masses were defined by the criteria of Silverman et al. [15].

**Table 1:** Patient demographics for each site and total

Variable	*N*	Overall *N* = 193^a^	Site 1 *N* = 153^a^	Site 2 *N* = 40^a^	*p*-value^b^
*Age (year)*	193	68.00 (59.00, 74.00)	67.00 (58.00, 73.00)	70.00 (65.00, 76.00)	**0.018**
*CEUS follow-up (months)*	193	35.10 (12.00, 70.00)	46.00 (24.00, 80.00)	9.50 (1.95, 32.70)	**< 0.001**
*CT follow-up (months)*	193	0.00 (0.00, 32.50)	0.00 (0.00, 41.00)	0.00 (0.00, 10.85)	0.072
*MRI follow-up (months)*	193	0.00 (0.00, 0.00)	0.00 (0.00, 0.00)	0.00 (0.00, 3.20)	**< 0.001**
*Total follow-up (months)*	192	36.00 (14.50, 74.00)	46.00 (24.00, 90.00)	12.35 (2.10, 35.10)	**< 0.001**
*Gender*	193				0.276
Female		78 (40.4%)	65 (42.5%)	13 (32.5%)	
Male		115 (59.6%)	88 (57.5%)	27 (67.5%)	
*Tumor side*	193				0.807
Left		98 (51%)	77 (50%)	21 (53%)	
Right		95 (49%)	76 (50%)	19 (48%)	
*Pathology size (cm)*	192	2.90 (2.20, 3.65)	3.00 (2.30, 3.80)	2.60 (2.00, 3.30)	0.080
*Type of renal tumor*	193				**0.026**
Cystic		17 (8.8%)	17 (11%)	0 (0%)	
Solid		176 (91%)	136 (89%)	40 (100%)	
*Type of treatment procedure*	193				**< 0.001**
Cryoablation		38 (19.7%)	13(8.5%)	25 (62.5%)	
Microwave ablation		4 (2.1%)	3 (2.0%)	1 (2.5%)	
Partial nephrectomy		135 (69.9%)	121(79.1%)	14 (35%)	
RFA		16 (8.3%)	16 (10.4%)	0 (0%)	

^a^Median (IQR) or Number (%)

^b^Wilcoxon rank sum test; Pearson’s Chi-squared test; Fisher’s exact test

FU: follow-up. CEUS: contrast enhancement ultrasound. CT: computed tomography. MRI: magnetic resonance imaging

The bold highlight the results that are statistically significant

Tumor histology included clear cell RCC (135, 69.9%), papillary RCC (27, 14.0%), chromophobe RCC (14, 7.3%), oncocytic RCC (8, 4.1%), multicystic RCC (3, 1.6%), eosinophilic RCC (1, 0.5%), RCC with rhabdoid features (1, 0.5%), and unknown due to FNA (4, 2.1%). Tumor types with histologic grades are listed in Table 2.

**Table 2:** Tumor type and grade for both sites

Tumor type and grade	Number
Clear Cell Carcinoma G1	48
Clear Cell Carcinoma G2	66
Clear Cell Carcinoma G3	21
Papillary Renal Cell Carcinoma G1	14
Papillary Renal Cell Carcinoma G2	9
Papillary Renal Cell Carcinoma G3	3
Papillary Renal Cell Carcinoma G4	1
Chromophobe Renal Cell Carcinoma G1	9
Chromophobe Renal Cell Carcinoma G2	3
Chromophobe Renal Cell Carcinoma G3	2
Oncocystic Renal Cell Carcinoma	8
Eosinophilic Renal Cell Carcinoma	1
Multicystic Renal Cell Carcinoma Low grade	3
Renal Cell Carcinoma with Rhabdoid features	1
FNA without tumor type or grade	4

**Fig. 2:**
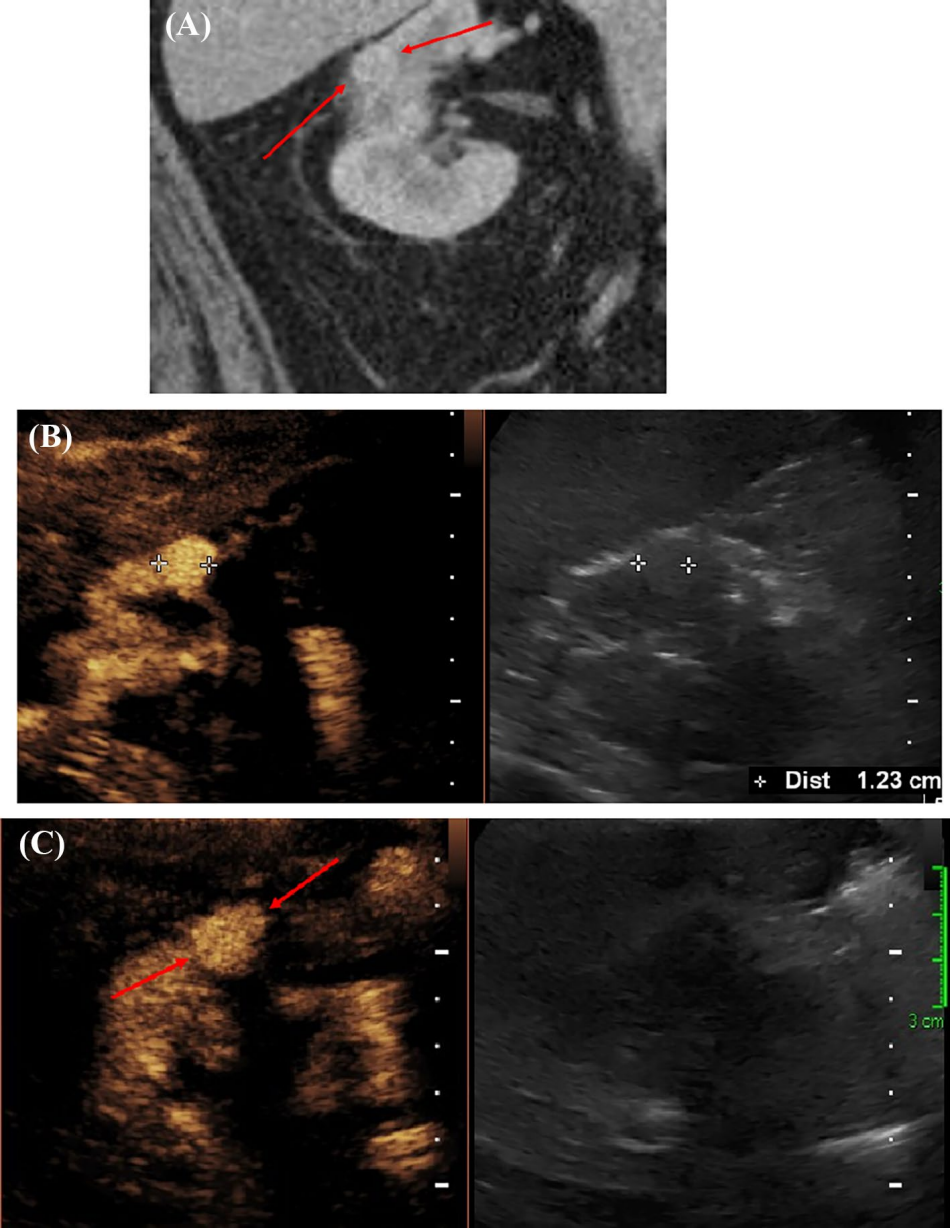
CEUS and CECT of a 67-year-old female with residual tumor post cryoablation for a clear cell renal carcinoma G2 pT1a. There is concordance with CECT (**A**, red arrows) and CEUS (**B**, measurement markers) with a 1.2 cm area of enhancement at the procedure site. The CEUS is a dual screen image with the contrast enhanced ultrasound on the left and a synthesized B-mode image on the right. The B-mode image is used to confirm localization and is not a diagnostic image. The patient was monitored and one year later the area of residual tumor had increased on CEUS to 1.8 cm (**C**, red arrows). The patient remains on surveillance monitoring

**Fig. 3:**
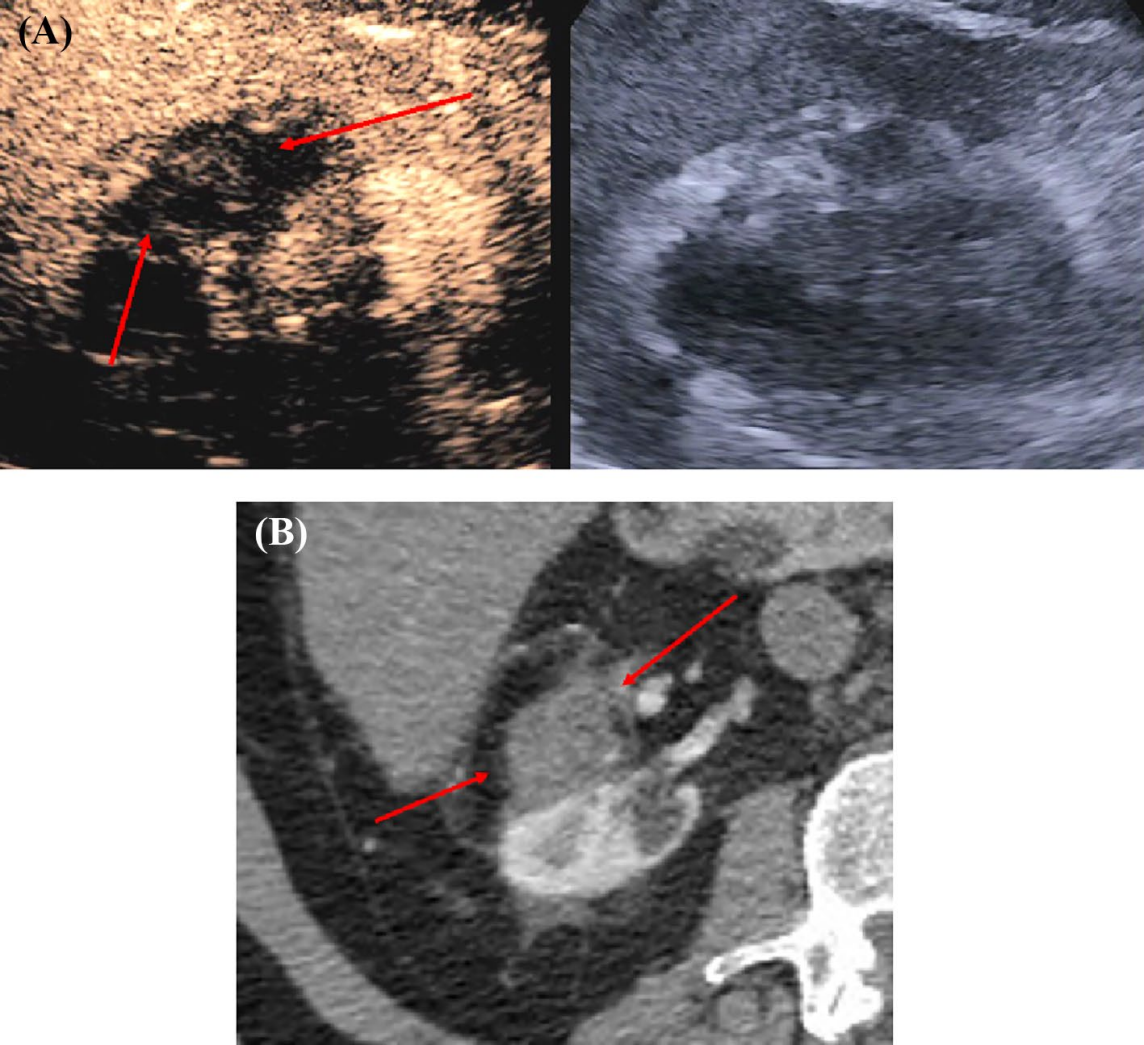
60-year-old male with residual tumor post RFA for a clear cell carcinoma G2 pT1a. one month post ablation. The CEUS (**A**) is positive with contrast within the ablation site (arrows) while the CECT (**B**) does not show any enhancement in the ablation site and is negative. The CEUS is a dual screen image with the contrast enhanced ultrasound on the left and a synthesized B-mode image on the right. The B-mode image is used to confirm localization and is not a diagnostic image. The patient is on surveillance monitoring and the enhancement on CEUS has not changed over a 1-year period

For all cases of CEUS positive the enhancement pattern was globular or round enhancement. For all procedures there was a total of 8.8% (17/193) positive residual or recurrence of tumor cases. This included 7.2% (11/153) for site 1 and 15% (6/40) for site 2. For combined sites for partial nephrectomy, the positive recurrence was 1.5% (2/135); RFA 43.7% (7/16); cryoablation 21.1% (8/38); MWA 0.0% (0/4). Based on follow-up procedures or continued enhancement over 1 year there was a 100% sensitivity (95% CI 81–100%) and 100% (95% CI 98–100%) specificity in detecting residual or recurrent tumor post nephron sparing procedures. Table 3 lists the results of the follow-up studies. Figure 2 is a 67-year-old female with residual tumor post cryoablation with concordant CECT and CEUS examinations. Figure 3 is a 60-year-old male with residual tumor post RFA where the CEUS is positive and the CECT is negative.

**Table 3:** Results of the CEUS examination by site and procedure

Site	procedure	CEUS +	CEUS −
1 + 2	All	17/193 (8.8%)	176/193 (91.2%)
1	RFA	7/16 (43.7%)	9/16 (56.3%)
1	Cyro ablation	3/13 (23.1%)	10/13(76.9%)
1	Microwave ablation	0/3 (0%)	3/3 (100%)
1	Partial nephrectomy	1/121 (0.8%)	120/121 (99.2%)
2	RFA	0	0
2	Cryoablation	5/25 (20.0%)	20/25 (80.0%)
2	Microwave ablation	0/1	1/1
2	Partial Nephrectomy	1/14 (7.1%)	13/14 (92.9%)

**Fig. 4:**
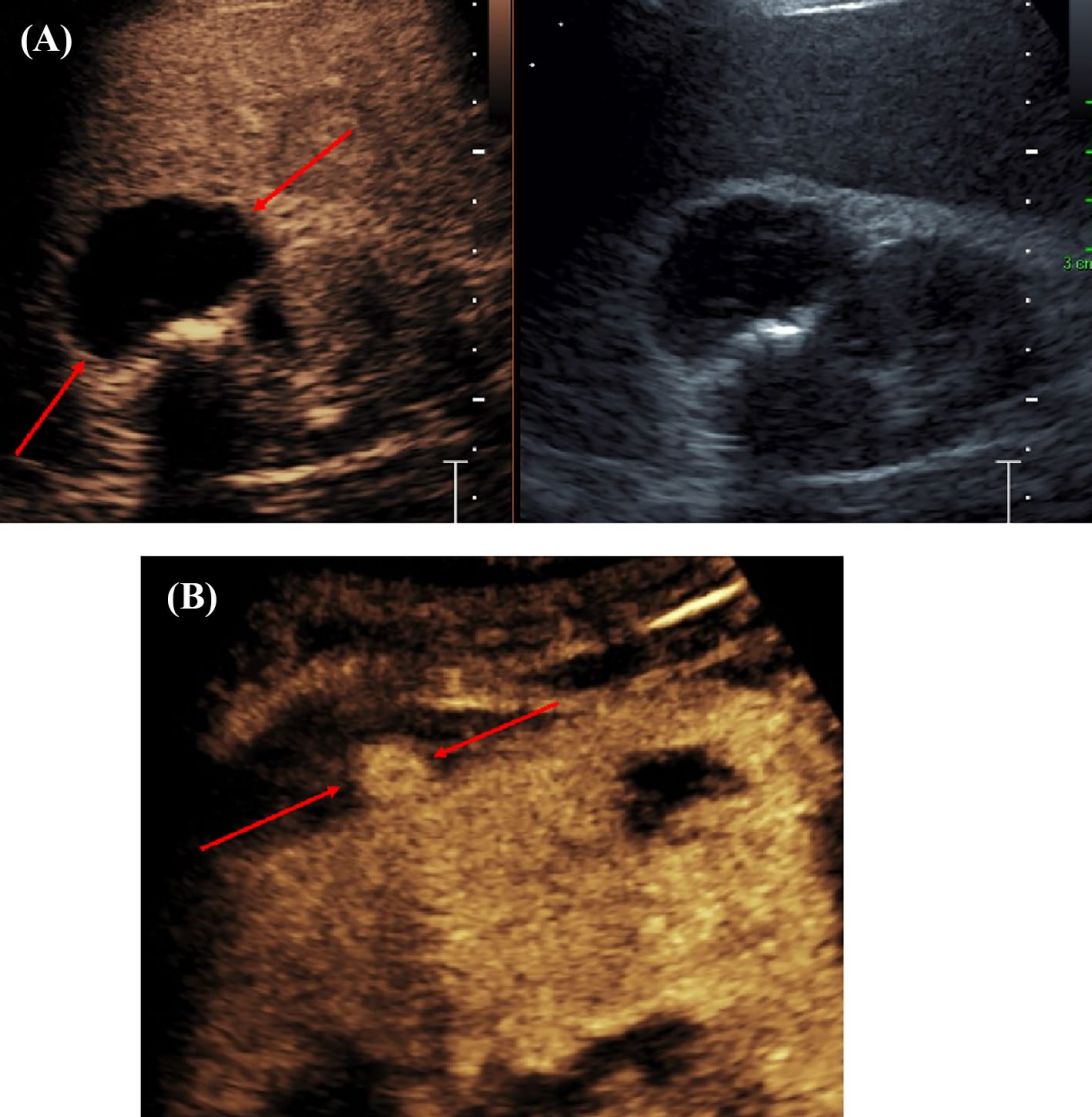
60-year-old male with a recurrence 6 years after partial nephrectomy for an oncocytic RCC. The CEUS is a dual screen image with the contrast enhanced ultrasound on the left and a synthesized B-mode image on the right. The B-mode image is used to confirm localization and is not a diagnostic image. The initial CEUS post partial nephrectomy (**A**) demonstrates no enhancement at the surgical site (red arrows). On follow-up CEUS 6 years (**B**) later there is a 1.0 cm enhancing nodule (red arrows). CECT or CEMRI were not performed. The patient remains in monitoring

**Fig. 5:**
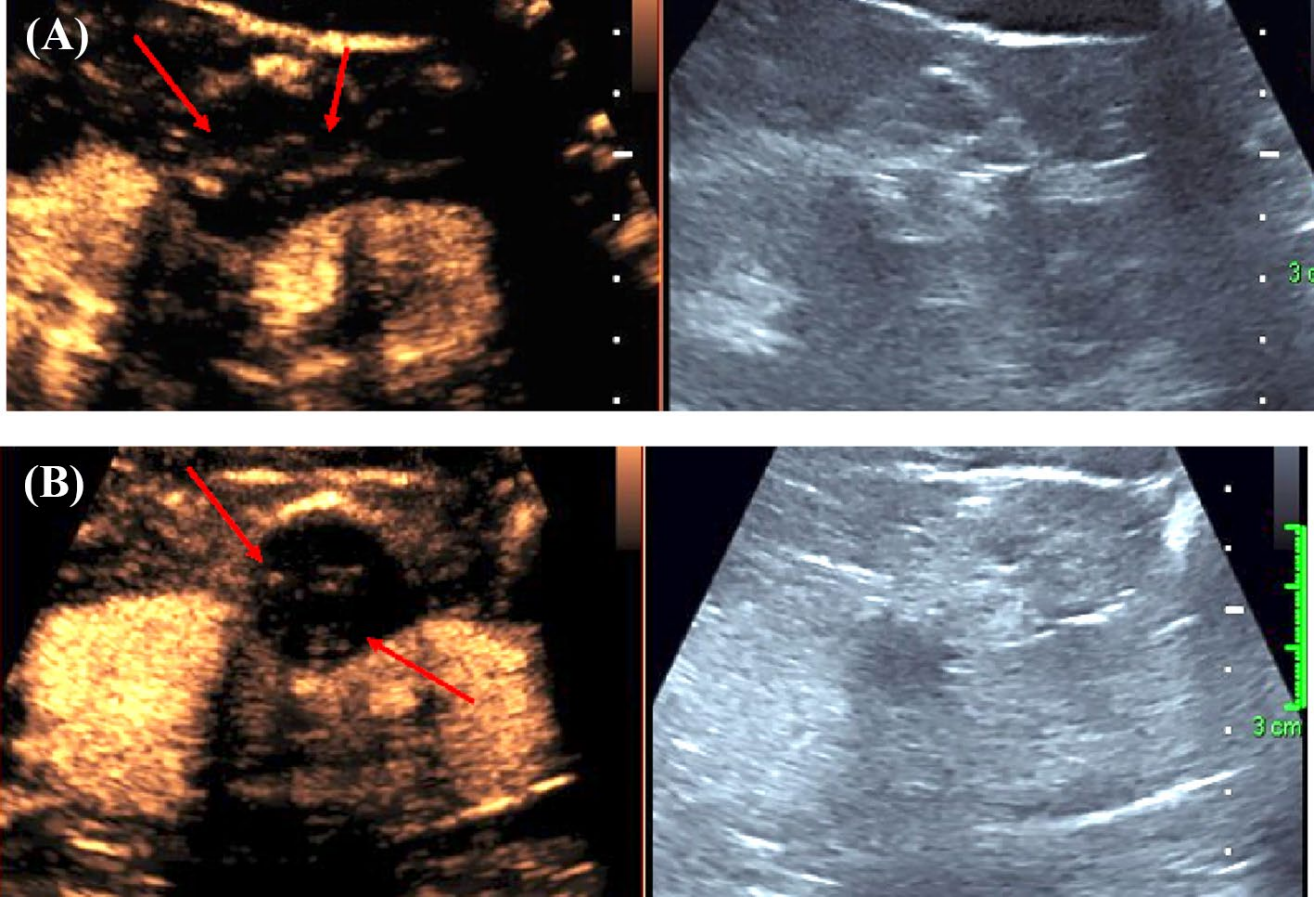
78-year-old female with enhancing septations in the ablation site post RFA of a clear cell carcinoma G1 pT1a. The CEUS is a dual screen image with the contrast enhanced ultrasound on the left and a synthesized B-mode image on the right. The B-mode image is used to confirm localization and is not a diagnostic image. On the initial post RFA CEUS (**A**) enhancing septations are noted (red arrows). Patient was followed and one year later (**B**) the enhancing septations (red arrows) are again noted and not significantly changed. The patient remains on monitoring. CECT or CEMRI were not performed

Of the 17 cases with abnormal post procedure enhancement, the two with partial nephrectomies had no further treatment; one is being monitored and the other expired with metastatic RCC. Of the 8 positive cryoablation cases 1 (12.5%) went on to partial nephrectomy, 2 (25.0%) remain on imaging surveillance monitoring, and 5 (62.5%) had repeat cryoablation with complete ablation documented on CEUS. Of the 7 positive cases post RFA, follow-up in 1 (14.3%) become positive after 6 years and 1 (14.3) became positive after 3 years of follow-up and both remain on monitoring, 3 (42.8%) remain on monitoring, and 1 (14.3%) had a repeat RFA with no residual tumor, 1 (14.3%) had a partial nephrectomy. Figure 4 depicts a 60-year-old male with a recurrence 6 years after partial nephrectomy. Figure 5 depicts a 78-year-old female with enhancing septations post RFA that persist for over 1 year. Table 4 lists the follow-up of the patients with positive residual tumor or recurrence.

**Table 4:** List of the positive cases, cell type and follow-up

Age	Sex	Site	Procedure	Histology	Follow-up
60	M	1	Partial Neph	Oncocytic RCC	No follow-up
70	M	1	Cyro	Clear Cell G1	Partial Nephrectomy
73	F	1	Cyro	Clear Cell G1	monitoring
67	F	1	Cyro	Clear Cell G1	Monitoring increased enhancement from 1.2 to 1.8 cm in 1 year
60	M	1	RFA	Clear cell G2	Turned + after 6 years
80	M	1	RFA	Clear cell G2	Repeat RFA
78	F	1	RFA	Clear cell G1	Monitoring – no change in enhancement
80	F	1	RFA	Clear Cell G1	Monitoring – now metastatic breast cancer
77	M	1	RFA	Clear Cell G1	Monitoring
77	M	1	RFA	Papillary RCC G1	Turned + after 3 years monitoring
73	M	1	RFA	Clear Cell G1	Partial nephrectomy
88	F	2	Cyro	Necrotic Tissue, Presumed Clear cell	Repeat Cryoablation, Negative
71	M	2	Cyro	Renal Cell Neoplasm with Clear Cell Features	Repeat Cryoablation, Negative
70	M	2	Cyro	Clear Cell Renal Carcinoma, Grade 2	Repeat Cryoablation, Negative
76	M	2	Cyro	Clear Cell Papillary Neoplasm	Repeat Cryoablation, Negative
69	F	2	Cyro	Clear cell Renal Caner, Grade 1	Repeat Cryoablation, Negative
80	M	2	Partial Neph	Clear Cell Renal Cancer, Grade 3	Metastatic RCC, Expired

Of the 17 positive cases 88.2%. (15/17) were cases with residual tumor on the first post procedure CEUS and 11.8% (2/7) had recurrence after an initial negative study.

Of the 193 cases 47.7% (92/193) had CECT scans and 7.3% (14/193) had CEMRI post procedure. CECT follow-up occurred from 1 month to 313 months and CEMRI follow-up occurred from 10 months to 120 months. There were 53.9% (104/193) cases that only had CEUS follow-up.

For site 1 there was at least 1 post procedure CECT in 48% (74/153) participants and for site 2 45% (18/40). For site 1 there was at least 1 post procedure CEMRI in 1.3% (2/153) participants and 30% (12/40) for site 2.

Of the 92 cases with both CECT and CEUS there was concordance in 96.7% (89/92) with 3 cases of CEUS positive and CECT negative findings. There was concordance of CEMRI and CEUS in 100% (14/14) cases. There were no cases with a positive CECT or CEMRI and a negative CEUS.

## Discussion

This retrospective two center study evaluating CEUS post nephron sparing procedures for RCC found a residual tumor/recurrence rate of 8.8% and a sensitivity of 100% (95%CI 81–100%) and specificity of 100% (95%CI 98–100%) for CEUS based on concordance with CECT or CEMRI or long turn follow-up. There was 96.7% concordance with CECT and 100% concordance with CEMRI, with three positive CEUS and negative CECT. The study also found 2 cases of recurrence one after 3 years and one after 6 years confirming the recommendation of routine continued follow-up after a confirmed completely treated tumor.

CEUS has the advantage of no renal toxicity, excellent safety profile, ability to give multiple doses at one siting, excellent background subtraction, thin slice thickness and real-time imaging. CEUS can be used to monitor the procedure and determine immediately after procedure if there is residual tumor so additional intervention can be done at the same time [9]. To our knowledge this is the largest study to date with the longest imaging follow-up of surveillance of post nephron sparing procedures. The study included multiple types of RCCs of sizes varying from 1.4 cm to 7.1 cm. Garbajs et al. [16] evaluated both CECT and CEUS for 20 patients up to 17 months. There was complete response in 64.3% of participants, residual tumor in 28.6% and progression in 7.1% given the assumptions without a reference standard. There was complete concordance between CEUS and CECT in the detection of residual enhancement when both were performed at the same CECE. Guo et al. [17] evaluated CEUS after radiofrequency ablation (RFA) in 31 patients. Within one month of the procedure, CEUS demonstrated high sensitivity (88.9%) and specificity (100%) for detecting residual tumor compared with CECT or CEMRI. Recurrence detection by CEUS matched CT or MRI at 100% sensitivity and specificity. There is a prospective on-going trial evaluating CEUS fused to pre-ablation CT or MRI performed within 4 weeks post-ablation. Interim analysis of 50 pts showed CEUS correlated perfectly (100% agreement) with reference CECT or CEMRI for recurrence detection, with good localization of ablation margins [18]. Vovdenko et al. [19] performed a meta-analysis of 12 trials using CEUS with CT or MRI as the reference standard. Within 6 weeks post-ablation, the pooled sensitivity for complete focal treatment was 90.2%, specificity 99.3%. Beyond 6 weeks, sensitivity 95.3%, specificity 97.6%. The negative predictive value was higher (> 98%).

The results of this study are slightly better than the previous smaller studies but consistent with a more recent study [17–19] with > 95% sensitivity and > 95% specificity for CEUS monitoring. Our study included 4 methods of nephron sparing procedures (RFA, MWA, cryoablation, and partial nephrectomy). There was a significant difference between the residual tumor or recurrence rate with partial nephrectomy having the lowest at 1.5% and RFA the highest rate at 43.7%. The method of nephron sparing procedure was decided by the referring physician. The efficacy is substantially influenced by the skill and experience of the performing physician and could vary greatly between sites. A recent meta-analysis [20] comparing the efficacy of ablative therapies for RCC found the efficacy for RFA were 96% (94–98; I^2^ = 73%) at 1 year, 95% (92–98; I^2^ = 77%) at 2 years, and 92% (88–96; I^2^ = 78%) at 5 years; for MWA were 97% (95–99; I^2^ = 74%) at 1 year, 95% (92–98; I^2^ = 77%) at 2 years, and 86% (75–94; I^2^ = 66%) at 5 years; and for cryoablations were 95% (93–96; I^2^ = 61%) at 1 year, 94% (91–96; I^2^ = 69%) at 2 years, and 90% (87–93; I^2^ = 74%) at 5 years. The RFA efficacy in our study was significantly worse than the literature suggests. Each site should assess their success rate for nephron sparing procedures and determine which technique is best for their site.

In this study there were two patients who had recurrence of their tumor at 3 years and 6 years after the treatment procedure confirming the American Urologic Association guideline recommendations for long term continued follow-up [12].

Many of patients with renal masses have renal insufficiency and CT and MRI contrast agents should be avoided. In addition, when the renal function could be decreased due to the ablation procedure, CEUS offers more safety for serial follow-up studies. Since these patients receive long term follow-up the use of CECT should be avoided due to the risks of the cumulative radiation dose.

This study has several limitations. First, we did not have a reference standard in all cases. Therefore, we do not report sensitivity and specificity. However, in all the case where we had biopsy and/or CECT there was exact concordance with CEUS. Contrast in the procedure site is the accepted standard for treatment failure on CECT and CEMRI. However, it has not been proven that enhancement with CEUS is treatment failure although there is no reason to assume CEUS is different than CECT or CEMRI. It is not confirmed that the presence of enhancement is always residual tumor and could represent fibrosis/inflammation. This would be the same for CECT and CEMRI. In fact, CEUS is an intravascular agent and does not extravasate which can occur with CECT or CEMRI most likely leading to more accurate results. In general, the vascularity due to inflammation/fibrosis decreases over time. Second, it was performed at two sites with extensive experience in performing renal CEUS. Sites that have less experience may not have similar results. Third, the renal vein and IVC status can be assessed with CEUS, however distant metastatic disease cannot be adequately evaluated with CEUS, and additional imaging is required for complete staging.

This study confirms that CEUS can be used to determine efficacy of nephron sparing procedures and that concurrent CECT or CEMRI may not be required for follow-up unless there is a question of the CEUS results or a technically inadequate study given the results of this study. CEUS may be more sensitive in detecting small amounts of enhancement with 3.2% (3/92) cases where CEUS was positive and CECT was negative. Continuous long-term follow-up is needed after these procedures as recurrences occurred at 3 years and 6 years in our study.

## Data Availability

No datasets were generated or analysed during the current study.
